# Khaki, Rank, and Recognition for Dispensers in Military Hospitals

**Published:** 1915-04-24

**Authors:** 


### THE EDITOR'S LETTER-BOX.

### Khaki, Rank, and Recognition for Dispensers in Military Hospitals.

*To the Editor of* The Hospital.

Sir,—On behalf of certificated dispensers now serving in military hospitals in place of the regular R.A.M.C. compounders, I should like to draw attention to the anomalous conditions now prevailing therein.

1. Any dispenser, whether certificated or not, is accepted, nevertheless the rate of pay is precisely similar for each.
2. Be his experience long or short, qualified or unqualified, there is no seniority, and all enter on the same basis.
3. Dispensers are treated partly on military lines and partly as civil subordinates, and no rank is given as in the medical profession.

I submit that certificated dispensers should be given rank and a higher rate of pay. This would be some measure of justice considering that as many as three to four hundred prescriptions are dispensed in one day, and that the accuracy and efficiency of the work of the hospitals largely depend upon the certificated and experienced man.

What would medical officers say if an unqualified medical man were given rank and pay similar to the qualified one? Dispensers have always been overlooked by the military authorities, and now is the time to give the certificated men khaki, rank, and recognition, since they are doing Government military work without even being granted the badge "On War Service."—Yours truly,

